# Impairment of renal function using hyperoncotic colloids in a two hit model of shock: a prospective randomized study

**DOI:** 10.1186/cc11161

**Published:** 2012-01-25

**Authors:** Tim Philipp Simon, Tobias Schuerholz, Lars Hüter, Michael Sasse, Florian Heyder, Wolfgang Pfister, Gernot Marx

**Affiliations:** 1 Department of Intensive Care and Intermediate Care, RWTH University Hospital Aachen, Pauwelsstrasse 30, 52074 Aachen, Germany; 2 Department of Intensive Care, Central Hospital Bad Berka, Robert-Koch-Allee 9, 99437 Bad Berka, Germany; 3 Department of Anesthesiology and Intensive Care Medicine, University of Jena, Erlanger Allee 101, 07747 Jena, Germany; 4 Department of Microbiology, Friedrich-Schiller University of Jena, Erlanger Allee 101, 07747 Jena, Germany

## Abstract

**Introduction:**

One of the therapeutic essentials in severe sepsis and septic shock is an adequate fluid replacement to restore and maintain circulating plasma volume, improve organ perfusion and nutritive microcirculatory flow. The type of solution to be used as a fluid replacement remains under discussion. The aim of the study was to evaluate the effects of clinically used fluid replacement solutions on renal function and inflammatory response.

**Methods:**

A total of 23 anesthetized and ventilated female German Landrace pigs were investigated over 19 hours using a two-hit model that combined hemorrhagic and septic shock. The septic shock was induced using an *Escherichia coli *laden clot placed into the abdominal cavity. Infusions of 6% hydroxyethylstarch 130/0.42 in acetate (6% HES 130), 4% gelatin in acetate (4% gelatin) and 10% hydroxyethylstarch 200/0.5 in saline (10% HES200) compared to Ringer's acetate (RAc) were used for fluid replacement to maintain a central venous pressure of 12 mmHg. Ringer's acetate was also used in the sham-treated group (SHAM).

**Results:**

At study end the cardiac output (10% HES200 143 ± 48 ml/kgBW; 6% HES130 171 ± 47 ml/kgBW; RAc 137 ± 32 ml/kgBW; 4% gelatin 160 ± 42 ml/kgBW), as well as mean arterial pressure did not differ between groups. N-acetyl-beta-D-glucosamidase was significantly higher in the hydroxyethylstarch 200 (157 ± 115 U/g creatinine; *P *< 0.05) group compared to hydroxyethylstarch 130 (24 ± 9 U/g creatinine), Ringer's acetate (2 ± 3 U/g creatinine) and SHAM (21 ± 15 U/g creatinine) at the study's end. Creatinine significantly increased by 87 ± 84 percent of baseline in the 10% HES200 group compared to RAc and 6% HES130. We demonstrated in the histology of the kidneys a significant increase in osmotic-nephrosis like lesions for 4% gelatin compared to RAc, 6% HES130 and SHAM. Urine output was lowest in the 10% HES200 and 4% gelatin group, however not significantly.

Interleukin(IL)-6 levels were significantly elevated in the 10% HES200 group (3,845 ± 1,472 pg/ml) two hours after sepsis induction compared to all other groups (6% HES130 1,492 ± 604 pg/ml; RAc 874 ± 363 pg/ml; 4% gelatin 1,623 ± 1,242 pg/ml).

**Conclusions:**

Despite similar maintenance of macrocirculation 6% hydroxyethylstarch 130/0.42 and Ringer's acetate significantly preserve renal function and attenuate tubular damage better than 10% hydroxyethylstarch 200/0.5 in saline.

## Introduction

In severe sepsis and septic shock, adequate fluid replacement is one of the essential therapeutic requirements to restore and maintain circulating plasma volume in order to improve organ perfusion and nutritive microcirculatory flow [1]. It has been shown that early goal-directed fluid resuscitation in patients with severe sepsis and septic shock is associated with improved outcome [2]. Recently, updated international guidelines indicate that there is no robust evidence favoring either colloids or crystalloids for fluid replacement in patients with sepsis or septic shock [3].

The question of which type of solution to use as a fluid replacement remains controversial [4]. In Europe, mostly artificial colloid solutions are used on the basis of gelatin and hydroxyethyl starches (HES). Adverse effects of colloid administration on renal function have spurred ongoing research into the pathological mechanisms [5].

In critical ill patients, acute renal dysfunction frequently deteriorates to renal failure due to hypovolemia, hypoxia, hypoperfusion and toxicity of drugs (for example, anti-infectives), as well as endothelial damage inducing pro-inflammatory response finally resulting in organ dysfunction. Thus, the evaluation of colloid associated effects in this context is difficult. Interestingly, the data on gelatin are limited.

Schortgen and colleagues showed that in severe sepsis and septic shock patients, there was no survival benefit and a higher rate of renal failure when using 6% HES 200/0.6-0.66 as compared to gelatin [6]. The negative impact of 10% HES 200/0.5 as compared to modified Ringer's lactate (RL) on renal function in severe septic/septic shock patients was also demonstrated in a recent randomized VISEP (efficacy of fluid substitution and insulin therapy in severe sepsis) trial [7].

A large prospective observational study including over 3,000 critically ill patients showed that in a length of ICU stay of more than 24 hours, sepsis, heart failure and haematological cancer were all significantly associated with the need for dialysis or haemofiltration therapy, but fluid replacement with HES was not [8].

So far, almost all colloids are dissolved in 0.9% NaCl or in 5% glucose. The use of these colloids may be associated with undesirable electrolyte disturbances or even acid-base derangements [9]. In experimental septic shock, fluid resuscitation using a balanced HES preparation resulted in significantly improved survival as compared with saline-based resuscitation [10].

Recent developments include the introduction of new formulations and newly available HES and gelatin products. HES in acetate with a low molecular weight (130 kD) and other balanced solutions have been developed. The profiles of these solutions appear to be useful for clinical situations where large amounts of fluid replacement solutions are administered [11].

Previously, we found that 10% HES 200/0.5 was associated with a significantly elevated inflammatory response in tubular monocytes. This pronounced pro-inflammatory effect of 10% HES 200/0.5 compared with 6% HES 130/0.42 caused more tubular damage than 6% HES 130/0.42 and RL [12].

Based on our *ex vivo *experiment we investigated effects of fluid solutions on renal function and inflammatory response in a porcine two-hit model that combines hemorrhagic and septic shock. We selected the commonly used balanced solutions 6% HES 130/0.42 in RAc and 4% modified gelatin in RAc, 10% HES 200/0.5 in 0.9% NaCl compared to Ringer's acetate (RAc).

## Materials and methods

### Animals

Female German Landrace pigs (*n *= 23; mean 28.1 ± 1.7 SD kg body weight (BW)) were used and standard procedures for laboratory animal care were followed. This study was approved by the institutional and local committee on the care and use of animals (Thüringer Landesamt für Lebensmittelsicherheit und Verbraucherschutz, Bad Langensalza, Germany).

### General anesthesia and surgical procedures

Animals were premedicated via intramuscular administration of ketamin and midazolam. Thereafter, general anesthesia was induced by intravenous injection of propofol (3 to 4 mg/kg BW) and the animals were orally intubated and placed in the supine position. General anesthesia was maintained with an infusion of propofol and sufentanil. Controlled pressure mode ventilation was chosen to ventilate the animals with an inspiratory oxygen fraction of 0.4, an inspiratory/expiratory ratio of 1:2, positive end-expiratory pressure (PEEP) set to 5 and a tidal volume of 8 to 10 ml/kg BW. Respiratory rate was set with the aim to maintain a PaCO_2 _of 35 to 46 mmHg. The core body temperature was kept at above 37.5°C with the use of a warming blanket. A transpulmonary thermodilution catheter, central venous line and the pulmonary artery catheter were placed as we described in detail in former papers [4]. None of the infusion systems were heparinized. In all animals, a midline laparotomy was performed and the left renal artery was exposed and a Transonic Doppler flow probe (Transonic Systems, Ithaca, NY, USA) was placed around the vessel to measure blood flow of the left renal artery (RBF).

### Escherichia coli fibrin clot

In this model, we used an *Escherichia coli *laden clot to induce septic shock. We used 7 to 9 × 10^9 ^colony forming units (CFU) per kg/BW. The clot was made from a sterile solution of porcine fibrinogen (Sigma-Aldrich Inc., St. Louis, MO, USA) (final volume was adjusted to the pig's weight). The clot was formed by adding thrombin from bovine plasma to the fibrinogen and *E. coli *solution and incubating for 30 minutes at room temperature.

### Hemodynamics

All intravascular pressure measurements were referenced to mid-chest level and values were obtained at end expiration. Mean arterial pressure (MAP), central venous pressure (CVP), mean pulmonary artery pressure (MPAP) and pulmonary artery occlusion pressure (PAOP) were recorded from calibrated pressure transducers (Edwards Lifesciences, Irvine, CA, USA) and values obtained at end-expiration.

Intrathoracic blood volume (ITBV) was calculated by the mean transit time approach (transpulmonary thermo dilution technique) using a PiCCO^® ^System (PULSION Medical Systems SE, Munich, Germany).

Cardiac output (CO) was determined by thermodilution with a CO computer (Vigilance^®^; Edwards Lifesciences Ltd, Newbury, UK).

Systemic oxygen delivery (DO_2_), oxygen extraction ratio (OE) and consumption (VO_2_) were calculated from hemoglobin, oxygen saturation, PaO_2 _and cardiac output according to standard formulae.

### Laboratory

Blood gas analyses were measured using a standard blood gas oximetry system (ABL 625; Radiometer, Copenhagen, Denmark) with a co-oximeter. Sodium, potassium, chloride, creatinine and hematocrit (Hct) were determined using standard laboratory techniques. We calculated the anion gap with the formula: anion gap = ((Na^+^)+(K^+^))-((Cl^-^)+(HCO_3_^-^)).

Colloid osmotic pressure (COP) was analyzed using a membrane colloid oncometer with a 20 kD semi-permeable membrane (BMT 921, Delta-Pharma, Pfullingen, Germany). From urine specimen, N-acetyl- beta-D-glucosamidase (NAG; analyzed by a spectrophotometric method; Hoffmann-La Roche, Basel, Switzerland), an approved sensitive marker of lysosomal tubular damage [10], was measured. Creatinine clearance was measured as an estimate of glomerular filtration rate (ClCrea = Ucrea × Uvol/Pcrea × duration of urine collection period; where Ucrea = urine creatinine concentration; Uvol = urine volume during the collection period; Pcrea = serum creatinine concentration). The duration of urine collection period was 240 minutes.

Cumulative fluid balance was calculated as fluid input minus urinary output during the entire study period. Body weight of animals was measured directly before experimentation.

### Flowmetry

Left renal arteries were identified close to their origins at the aortas. A pre-calibrated ultrasonic transit time flow probe (Transonic Systems Inc., Ithaca, NY, USA) was placed around the vessels and connected to an ultrasound blood flow meter (T207; Transonic Systems Inc.).

### Cytokines

Levels of interleukin 6 (IL-6), interleukin 10 (IL-10) and tumor necrosis factor alpha (TNF-α) in plasma were assessed by an enzyme-linked immunosorbent assay using commercially available kits specific for pigs (IL-6; IL-10; TNF-α: BioSource, Solingen, Germany). Measurements were made according to the manufacturer's guidelines and blood samples were collected additionally two and four hours after sepsis induction.

### Histological analysis

Biopsies of the kidney were taken directly after euthanasia of the animals, fixed in 4% formaldehyde and embedded in paraffin. Sections (4 μm) were cut and stained with hematoxylin and eosin (H&E). Renal morphology was assessed semiquantitatively using the following four criteria of tubulointerstitial renal injury: acute tubular necrosis (ATN), that is, dilatation of tubuli with flattening or loss of the tubular epithelium, interstitial bleeding (IBl), glomerulo sclerosis (GSl) and osmotic-nephrosis like lesions (OL) of the tubuli (see also Additional files 1, 2, 3). One experienced investigator blinded to the groups scored each variable using a semiquantitative scoring system (0 to 4) for each criterion in 20 randomly sampled visual fields per animal: 0 (absent), 1 (0 to 25%), 2 (25 to 50%), 3 (50 to 75%) or 4 (more than 75%) [10].

### Experimental protocol

During the surgical procedure, animals received 10 ml/kg BW/h RAc (Serumwerke Bernburg AG, Bernburg, Germany). Hemorrhagic shock was induced by bleeding the animals via the carotid artery catheter. Animals were bled until half of the baseline MAP was reached, resulting in a blood loss of 32.8 ± 3.8 ml/kg BW per animal. Hemorrhagic shock was maintained for 45 minutes. Thereafter, animals were randomly allocated to receive either fluid therapy with RAc (*n *= 5), 4% gelatin in RAc (4% gelatin) (Gelafusal^®^, Serumwerke Bernburg AG, Bernburg, Germany) (*n *= 5), 6% HES with a molecular weight of 130 kD and a molar substitution of 0.42 in RAc (HES 130) (Vitafusal^®^, Serumwerke Bernburg AG, Bernburg, Germany) (*n *= 5), 10% HES with a molecular weight of 200 kD and a molar substitution of 0.5 in 0.9% sodium chloride (HES 200) (Infukoll^®^, Serumwerke Bernburg AG AG, Bernburg, Germany) (*n *= 5), compared to a non-shock, sham-operated control group (SHAM, *n *= 3) receiving RAc. Fluid replacement was performed while maintaining baseline MAP for six hours. In addition, four hours after cessation of the hemorrhagic shock, the blood collected during hemorrhagic shock was retransfused.

Six hours after hemorrhagic shock, the infected clot was placed into the abdominal cavity to induce septic shock. SHAM group received a non-infected clot.

**Figure 1:**
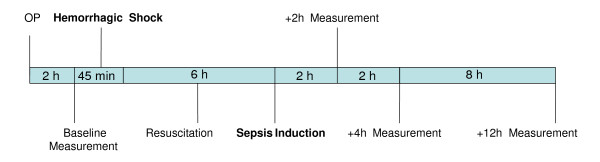
**Schedule of the two-hit model**.

Two hours after the induction of septic shock, the hemodynamic treatment scheme aimed at maintaining a CVP of 12 mmHg was initiated. Fluid therapy during this second shock was the identical randomized solution that was administered during hemorrhagic shock; hence, each animal received only one study solution. CVP was measured continuously and a gradual continuous fluid resuscitation was carried out. After induction of sepsis, animals were treated for another 12 h. Measurements were performed at baseline, before induction of septic shock (6 h after resolution of hemorrhagic shock) and 12 h after induction of septic shock (Figure 1).

### Statistical analysis

Data were analyzed using SPSS 16.0 for Windows (IBM Statistics, Ehningen, Germany) and all results are presented as mean ± standard deviation (SD). After verifying normal data distribution (skewness < 1.5) [13], effects of fluid replacement solution were statistically analyzed by analysis of variance (ANOVA) for repeated measurements. Bonferroni's correction was applied for multiple comparisons, following ANOVA for each single time point. Differences were considered significant at *P *< 0.05. Data are presented at baseline (BL), 6 h after hemorrhagic and before septic shock (SL) and 12 h after sepsis induction (12 h sepsis).

A three-fold increase in beta-NAG was regarded as clinically relevant. A total sample size of at least 23 animals was necessary in order to achieve a power of at least 80%.

## Results

In all animals, study protocol was completed. All animals reached the predefined target in hemorrhagic shock and fluid resuscitation. There were no differences in blood loss between the groups.

### Hemodynamics

Hemodynamic parameters are summarized in Table 1. Baseline values were comparable in all groups. Twelve hours after sepsis induction CO, as well as MAP, CVP and PAOP, did not differ between groups. MPAP increased significantly in the HES200 group 12 h after sepsis induction compared to the RAc, 6% HES130 and SHAM group (Table 1).

**Table 1:** Hemodynamic parameters and fluid balance

		6% HES 130	10% HES 200	RAc	4% gelatin	SHAM
**HR, bpm**	**Baseline**	93 ± 14	91 ± 10	89 ± 12	91 ± 9	98 ± 8
	**Before sepsis**	95 ± 5	96 ± 22	106 ± 20	92 ± 13	98 ± 11
	**12 h after sepsis**	140 ± 19	158 ± 19 ‡	127 ± 18	151 ± 32 ‡	93 ± 2
**MAP, mm Hg**	**Baseline**	90 ± 1	91 ± 2	92 ± 2	91 ± 2	90 ± 1
	**Before sepsis**	91 ± 3	98 ± 8	91 ± 1	93 ± 4	91 ± 3
	**12 h after sepsis**	86 ± 11	70 ± 21	59 ± 5	71 ± 26	96 ± 7
**CVP, mm Hg**	**Baseline**	7 ± 1	6 ± 1	7 ± 1	7 ± 1	6 ± 1
	**Before sepsis**	6 ± 2	9 ± 2	8 ± 1	8 ± 1	6 ± 1
	**12 h after sepsis**	12 ± 0	12 ± 0	12 ± 0	12 ± 0	12 ± 0
**PAOP, mm Hg**	**Baseline**	8 ± 1	7 ± 1	8 ± 1	7 ± 1	7 ± 2
	**Before sepsis**	8 ± 2	8 ± 1	10 ± 2	9 ± 2	8 ± 1
	**12 h after sepsis**	11 ± 3	12 ± 1	11 ± 2	10 ± 1	12 ± 1
**MPAP, mm Hg**	**Baseline**	15 ± 2	16 ± 3	19 ± 1	17 ± 2	18 ± 2
	**Before sepsis**	17 ± 2	20 ± 4	20 ± 1	19 ± 3	21 ± 1
	**12 h after sepsis**	33 ± 9	45 ± 3 *,§,‡	26 ± 4	41 ± 3 §,‡	21 ± 4
**CO, ml/kgBW**	**Baseline**	102 ± 20	109 ± 17	110 ± 15	98 ± 8	110 ± 7
	**Before sepsis**	118 ± 13	133 ± 37	134 ± 28	137 ± 26	126 ± 19
	**12 h after sepsis**	171 ± 47	143 ± 48	137 ± 32	160 ± 42	120 ± 28
**Hct, %**	**Baseline**	29 ± 1	31 ± 2	28 ± 2	30 ± 3	30 ± 1
	**Before sepsis**	26 ± 1	25 ± 4	26 ± 2	24 ± 4	30 ± 4
	**12 h after sepsis**	28 ± 4	30 ± 4	27 ± 3	25 ± 5	29 ± 4
**COP, mm Hg**	**Baseline**	11.5 ± 0.9	11.4 ± 2.4	11.1 ± 2.2	11.2 ± 1.0	12.2 ± 1.1
	**Before sepsis**	11.8 ± 1.4	13.6 ± 1.7	9.9 ± 1.1	12.5 ± 0.9	10.9 ± 0.3
	**12 h after sepsis**	10.0 ± 1.7	14.6 ± 1.8 *;§;‡	8.1 ± 1.5	15.1 ± 1.5 *;§;‡	10.3 ± 0.4
**Fluid input, ml/kg BW**	**Baseline**	-	-	-	-	-
	**Before sepsis**	62 ± 11	45 ± 12	140 ± 41║	62 ± 14	5 ± 7
	**12 h after sepsis**	213 ± 41	120 ± 35	556 ± 230║	178 ± 36	110 ± 26
**Fluid balance, ml/kg BW**	**Baseline**	-	-	-	-	-
	**Before sepsis**	39 ± 3	28 ± 10	93 ± 25║	45 ± 17	18 ± 11
	**12 h after sepsis**	149 ± 30	75 ± 33	335 ± 94║	109 ± 28	68 ± 43

Hemodynamics and Fluid balance in the five groups. Data are presented as mean ± SD at baseline, 6 h after hemorrhagic shock and before sepsis induction (before sepsis) and 12 h after sepsis induction. BW, body weight; CO, cardiac output; COP, colloid osmotic pressure; CVP, central venous pressure; Hct, hematocrit; HR, heart rate; MAP, mean arterial pressure; MPAP, mean pulmonary artery pressure; PAOP, pulmonary artery occlusion pressure.

* *P *≤ 0.05 vs. 6% HES130; § *P *≤ 0.05 vs. RAc;;‡ *P *≤ 0.05 vs. SHAM; ║*P *≤ 0.05 vs. all other groups.

### Fluid balance and laboratory

Treatment with RAc led to a significantly higher fluid input and fluid balance in the RAc group as compared to all other groups after 12 h of sepsis. The degree of Hct was similar in all groups at baseline and 12 h after induction of sepsis (Table 1). COP was significantly higher in 10% HES200 and 4% gelatin group after 12 h of sepsis compared to 6% HES130 and RAc (Table 1).

### Inflammation

**Figure 2:**
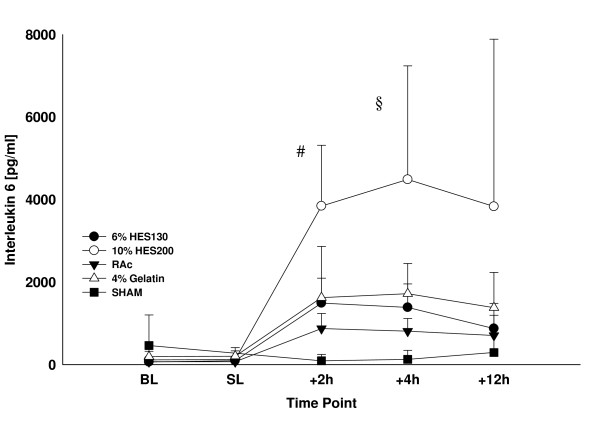
**Interleukin 6 levels over study period**. Data are presented as mean ± standard deviation in pg/ml at baseline (BL), 6 h after hemorrhagic shock and before sepsis induction (SL) and 2, 4 and 12 h after sepsis induction * *P *≤ 0.05 vs. 6% HES130, RAc und SHAM § = *P *≤ 0.05 vs. all other groups.

The inflammatory response was enhanced in all septic groups two hours after sepsis induction. IL-6 (Figure 2) was significantly higher in the 10% HES200 group compared to all other groups two hours after sepsis induction and TNF-α increased significantly in the 10% HES200 group (4,122 ± 1,417 pg/ml; *P *< 0.05) compared to all other groups (6% HES130 1,199 ± 1,237 pg/ml; 4% gelatin 1,939 ± 1,691 pg/ml; RAc 1,101 ± 498 pg/ml; SHAM 285 ± 385 pg/ml) two hours after sepsis induction (Table 2).

**Table 2:** TNF-α levels

	6% HES 130	10% HES 200	RAc	4% gelatin	SHAM
**BL**	131 ± 57	176 ± 92	172 ± 56	270 ± 214	226 ± 146
**Before sepsis**	183 ± 93	255 ± 241	125 ± 57	325 ± 210	95 ± 83
**2 h after sepsis**	1,199 ± 1,239	4,122 ± 1,216*	1,101 ± 498	1,938 ± 1,691	285 ± 386
**4 h after sepsis**	665 ± 384	2,396 ± 2,656	501 ± 215	746 ± 851	251 ± 364
**12 h after sepsis**	280 ± 75	673 ± 618	252 ± 64	437 ± 83	400 ± 326

Data are presented as mean ± standard deviation in pg/ml at baseline (BL), 6 h after hemorrhagic shock and before sepsis (before sepsis), 2 h, 4 h and 12 h after sepsis induction. * *P *≤ 0.01 vs. 6% HES130, RAc and SHAM

IL-10 was significantly elevated after 12 h of sepsis in the 10% HES200 group compared to 6% HES130, RAc and SHAM (Table 3).

**Table 3:** Measured and calculated parameters of oxygenation, hemodynamics, inflammation and acid-base metabolism

		6% HES 130	10% HES 200	RAc	4% gelatin	SHAM
**ITBV, ml/kgBW**	**Baseline**	21 ± 4	22 ± 4	23 ± 2	23 ± 4	23 ± 1
	**Before sepsis**	22 ± 2	25 ± 5	23 ± 1	25 ± 1	24 ± 2
	**12 h after sepsis**	24 ± 4	22 ± 5	22 ± 3	26 ± 2	24 ± 1
**DO_2_, ml/min/kgBW**	**Baseline**	12.9 ± 2.5	14.6 ± 1.6	13.5 ± 1.4	13.2 ± 1.2	14.7 ± 0.8
	**Before sepsis**	14.0 ± 3.1	12.5 ± 2.1	14.2 ± 1.2	15.0 ± 4.2	17.6 ± 0.6
	**12 h after sepsis**	17.7 ± 3.0	17.8 ± 7.8	15.3 ± 3.3	14.0 ± 2.5	15.4 ± 1.8
**OE %**	**Baseline**	39 ± 8	34 ± 2	36 ± 5	33 ± 6	31 ± 8
	**Before sepsis**	31 ± 15	32 ± 5	38 ± 6	30 ± 3	31 ± 5
	**12 h after sepsis**	37 ± 6	48 ± 8 ‡	35 ± 9	48 ± 10 ‡	27 ± 6
**VO_2 _ml/min kgBW**	**Baseline**	5.3 ± 1.0	5.3 ± 0.4	5.2 ± 1.2	4.4 ± 0.6	4.8 ± 1.2
	**Before sepsis**	4.2 ± 1.8	4.7 ± 0.8	6.1 ± 2.0	4.5 ± 1.3	5.7 ± 0.6
	**12 h after sepsis**	7.4 ± 2.5	8.7 ± 4.5	5.2 ± 1.1	7.1 ± 1.8	4.2 ± 0.6
**SvO_2_, %**	**Baseline**	65 ± 7	69 ± 2	67 ± 3	69 ± 7	71 ± 7
	**Before sepsis**	74 ± 13	69 ± 7	68 ± 8	70 ± 7	70 ± 7
	**12 h after sepsis**	62 ± 12	46 ± 9 ‡	60 ± 14	46 ± 12 ‡	75 ± 4
**pHa**	**Baseline**	7.49 ± 0.04	7.50 ± 0.03	7.50 ± 0.05	7.50 ± 0.01	7.53 ± 0.02
	**Before sepsis**	7.54 ± 0.03	7.51 ± 0.02	7.51 ± 0.04	7.52 ± 0.02	7.52 ± 0.03
	**12 h after sepsis**	7.48 ± 0.04	7.27 ± 0.12 *	7.47 ± 0.07	7.38 ± 0.01	7.49 ± 0.02
**BE, mmol/l**	**Baseline**	6 ± 2	6 ± 1	7 ± 3	7 ± 1	7 ± 1
	**Before sepsis**	8 ± 1	7 ± 1	8 ± 2	9 ± 1	7 ± 1
	**12 h after sepsis**	6 ± 1	-3 ± 5 *	7 ± 3	4 ± 6	7 ± 1
**PaCO_2_, mm Hg**	**Baseline**	39 ± 3	38 ± 3	40 ± 3	39 ± 2	38 ± 2
	**Before sepsis**	36 ± 3	38 ± 2	39 ± 2	39 ± 2	38 ± 3
	**12 h after sepsis**	40 ± 6	46 ± 5	43 ± 5	46 ± 6	38 ± 2
**Interleukin 10, pg/ml**	**Baseline**	-	-	-	-	-
	**Before sepsis**	-	-	-	-	-
	**12 h after sepsis**	88 ± 197	411 ± 245*	14 ± 31	296 ± 95	0 ± 0

Data are presented as mean ± SD at baseline, 6 h after hemorrhagic shock and before sepsis induction (before sepsis) and 12 h after sepsis induction. BW, body weight; DO_2_, systemic oxygen delivery; HES, hydroxyethylstarch; ITBV, intrathoracic blood volume; OE, oxygen extraction ratio; PaCO_2_, arterial CO_2_; pHa, arterial pH; BE, base excess; SvO_2_, mixed venous oxygen saturation; VO_2_, oxygen consumption. * *P *≤ 0.05 vs. 6% HES130, RAc and SHAM; ‡ *P *≤ 0.05 vs. SHAM.

### Systemic oxygenation

Results of systemic oxygenation are summarized in Table 3. SvO_2 _was comparable at baseline in all groups. Twelve hours after sepsis induction a significantly lower SvO_2 _was observed in the 4% gelatin and 10% HES200 group compared to SHAM (Table 3). Oxygen extraction ratio (OE) increased significantly in the 4% gelatin and 10% HES200 group compared to SHAM.

### Renal function and histology

Electrolyte levels at baseline were comparable in all groups (Table 4). There were no differences in sodium levels between groups. Chloride levels were significantly lower in the 4% gelatin group compared to 10% HES200 and RAc after 12 h of sepsis.

**Table 4:** Renal perfusion and function

		6% HES 130	10% HES 200	RAc	4% gelatin	SHAM
**Left renal artery flow, ml/kgBW/min**	**Baseline**	7.0 ± 2.9	7.5 ± 2.9	6.1 ± 2.2	6.8 ± 0.4	8.3 ± 3.7
	**Before sepsis**	5.3 ± 3.8	6.8 ± 2.6	4.6 ± 1.5	6.4 ± 0.5	8.9 ± 5.0
	**12 h after sepsis**	9.6 ± 3.7	4.0 ± 3.1	5.0 ± 1.9	6.7 ± 2.0	11.4 ± 8.3
**Crea as percentage of baseline, %**	**Baseline**	-	-	-	-	-
	**Before sepsis**	4 ± 5	12 ± 5 ‡	10 ± 6	14 ± 6 ‡	-2 ± 2
	**12 h after sepsis**	-13 ± 7	87 ± 84**; +	-4 ± 13	26 ± 13	-4 ± 13
**Crea clearance, ml/min**	**Baseline**	136 ± 24	130 ± 87	108 ± 94	101 ± 46	117 ± 13
	**Before sepsis**	98 ± 47	57 ± 36	57 ± 51	48 ± 17	78 ± 10
	**12 h after sepsis**	97 ± 15	13 ± 14 *	76 ± 23	38 ± 8 ‡; +	98 ± 48
**Urine output, ml/kgBW/h**	**Baseline**	-	-	-	-	-
	**Before sepsis**	-	-	-	-	-
	**12 h after sepsis**	3.3 ± 1.3	0.5 ± 0.3	11.5 ± 12.3	1.1 ± 0.2	2.2 ± 1.9
**Cl, mmol/l**	**Baseline**	100 ± 1	100 ± 2	99 ± 2	100 ± 2	100 ± 1
	**Before sepsis**	100 ± 1	103 ± 2	100 ± 1	98 ± 2	101 ± 2
	**12 h after sepsis**	103 ± 2	111 ± 2	109 ± 9	98 ± 2 **; ║	101 ± 2
**Na, mmol/l**	**Baseline**	135 ± 1	135 ± 2	136 ± 2	136 ± 1	136 ± 1
	**Before sepsis**	136 ± 2	138 ± 2	137 ± 3	138 ± 2	137 ± 1
	**12 h after sepsis**	136 ± 2	140 ± 3	144 ± 11	138 ± 3	136 ± 2
**K^+^, mmol/l**	**Baseline**	4.5 ± 0.4	4.4 ± 0.3	4.5 ± 0.2	4.4 ± 0.1	4.4 ± 0.2
	**Before sepsis**	3.7 ± 0.2	3.8 ± 0.3	4.0 ± 0.2	3.9 ± 0.2	4.4 ± 0.1
	**12 h after sepsis**	3.9 ± 0.5	5.6 ± 1.7	4.0 ± 0.3	4.9 ± 1.1	3.8 ± 0.1
**Aniongap mmol/l**	**Baseline**	10.2 ± 0.8	10.0 ± 0.3	9.9 ± 1.9	10.4 ± 0.5	10.4 ± 1.4
	**Before sepsis**	9.6 ± 0.3	9.2 ± 1.4	9.8 ± 1.3	11.4 ± 1.0	9.4 ± 1.4
	**12 h after sepsis**	6.8 ± 0.7	10.9 ± 4.2	8.2 ± 1.2	15.4 ± 4.2**;+	9.5 ± 0.2

Data are presented as mean ± SD at baseline, 6 h after hemorrhagic shock and before sepsis induction (before sepsis) and 12 h after sepsis induction.

BW, body weight; Cl, chloride; Crea, creatinine; K^+ ^, potassium; Na, sodium.

* *P *≤ 0.01 vs. 6% HES130, RAc and SHAM; ** *P *≤ 0.05 vs. RAc; + *P *≤ 0.05 vs. 6% HES130; ‡ *P *≤ 0.05 vs. SHAM; ║ *P *≤ 0.05 vs. 10% HES200.

Serum creatinine (as percent of baseline, a negative value means creatinine is decreasing compared to baseline) was significantly higher in the 10% HES200 group compared to RAc and 6% HES130 12 h after sepsis, and creatinine clearance was significantly lower in the 10% HES200 and the 4% gelatin group compared to 6% HES130 and SHAM 12 h after sepsis. Urine output at study end was lower in the 10% HES200 group compared to all other groups (Table 4).

**Figure 3:**
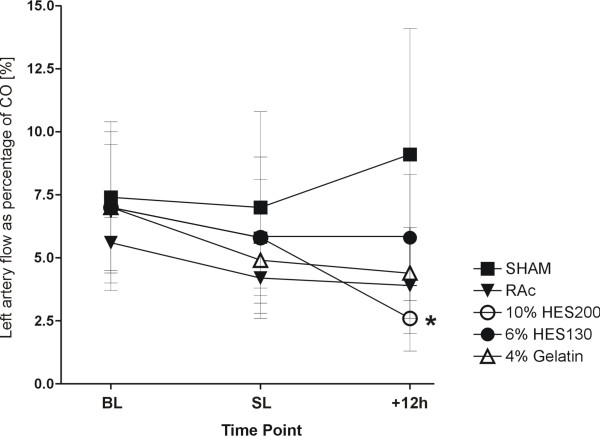
**Left renal artery flow as percentage of CO at baseline**. Data are presented as mean ± standard deviation at baseline (BL), 6 h after hemorrhagic shock and before sepsis induction (SL) and 12 h after sepsis induction * = *P *≤ 0.05 vs. SHAM.

The renal artery flow of the left kidney as percentage of CO 12 h after sepsis was lower, but not significant, in the 10% HES200 group compared to all other septic groups, and significantly lower compared to the SHAM group (Figure 3).

**Figure 4:**
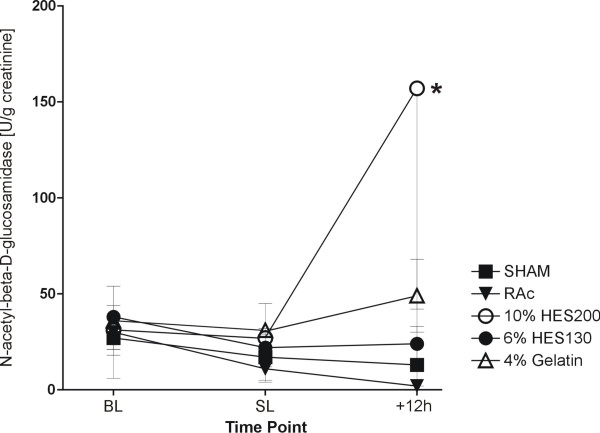
**N-acetyl-beta-D-glucosamidase in urine as a marker of lysosomal tubular damage**. Data are presented as mean ± standard deviation in Units per gram creatinine at baseline (BL), 6 h after hemorrhagic shock and before sepsis induction (SL) and 12 h after sepsis induction * = *P *≤ 0.05 vs. 6% HES130, RAc und SHAM.

Baseline values of NAG in urine (in units per gram creatinine (U/g creatinine)) were comparable in all groups. Fluid replacement with 10% HES200 led to a significantly higher NAG at study end compared to 6% HES130, RAc and SHAM (Figure 4).

The histological analyses are presented in Table 5. They showed a significant increase in OL for 4% gelatin compared to RAc, 6% HES130 and SHAM. ATN was significantly higher in 10% HES200 compared to SHAM. Also IBl was significantly higher in 10% HES200 compared to RAc, 6% HES130 and SHAM. There was a non- significant increase in GSl in the 10% HES200 group compared to all other groups (Table 5).

**Table 5:** Histopathological scoring of kidneys

	6% HES 130	10% HES 200	RAc	4% gelatin	SHAM
**Ol**	1.0 ± 0.9	1.5 ± 1.0	0.3 ± 0.3	2.6 ± 0.5*	0.5 ± 0.0
**ATN**	1.8 ± 1.1	2.2 ± 0.4‡	0.8 ± 0.3	2.6 ± 0.5‡**	0.3 ± 0.6
**IBl**	0.4 ± 0.5	1.6 ± 0.9*	0.3 ± 0.5	0.6 ± 0.4	0.0 ± 0.0
**GSl**	1.3 ± 1.0	1.4 ± 0.5	0.6 ± 0.3	1.1 ± 0.5	0.7 ± 0.3

Histopathological scoring of osmotic nephrosis-like lesions (OL), acute tubulus necrosis (ATN), interstitial bleeding (IBl) and glomerular sclerosis (GSl). * *P *≤ 0.01 vs. 6% HES130, RAc and SHAM ** *P *≤ 0.05 vs. RAc; ‡ *P *≤ 0.05 vs. SHAM

Data are presented as mean ± standard deviation.

## Discussion

The most important result of the present study is that in pigs after hemorrhagic and consecutive septic shock, 6% HES 130/0.42 in RAc and RAc solution are more effective in preserving renal function. Furthermore, we demonstrated a significant higher NAG and ATN indicating a more pronounced tubular damage using hyperoncotic 10% HES 200/0.5 in 0.9% saline.

### Hemodynamics

In macro-circulation there were no differences between the fluid replacement solutions tested. There were significant differences in oxygen extraction and SvO2 between 10% HES200 and 4% gelatin compared to SHAM and no differences between septic groups.

### Renal function

Sepsis is often associated with impaired renal function. The effect of HES infusion on renal function in sepsis patients has spurred ongoing research on the underlying pathology.

Schortgen and colleagues showed HES to be an independent risk factor for acute renal failure in severe sepsis [6]. The methodology of this study has been questioned, although it was randomized and controlled [9,14]. Recently, in a German multicenter randomized controlled trial (efficacy of fluid substitution and insulin therapy in severe sepsis (VISEP) study), it was shown that the use of 10% HES 200/0.5 compared with lactated Ringer's solution (RL) in patients with severe sepsis or septic shock is associated with an increased need for renal replacement therapy. Furthermore, the cumulative dosage of 10% HES200/0.5 was significantly correlated with the need for renal replacement therapy [7]. In contrast, a recent large prospective observational study of over 3,000 critically ill patients, showed that in those patients with an ICU stay of more than 24 hours, sepsis, heart failure and hematological cancer were all significantly associated with the need for dialysis or hemofiltration therapy, but fluid replacement with HES was not [14]. Comparing this result with the data of the VISEP study, one important difference is the total amount of administered HES. In the VISEP study, patients received HES for up to 21 days with a median cumulative dose of 70.4 ml/kg (interquartile range: 33.4 to 144.2 ml/kg), whereas in the observational study, the median total amount of HES per patient was lower (1,000 ml, interquartile range: 500 to 2,250 ml corresponding to a cumulative dose of less than 15 ml/kg).

These data as well as the results of our study on HES and renal function suggest that the degree of functional impairment might depend on the HES formulation used. In our study, in comparison to 6% HES130 and RAc, the use of 10% HES200 was associated with a significant lower creatinine clearance and higher creatinine levels. Furthermore, significantly higher NAG levels in the urine indicated a higher degree of tubular damage in this group.

Our results are in line with a recent pharmacokinetic study of healthy volunteers demonstrating that repeated administration of 6% HES 130/0.42 shows no accumulation compared to 10% HES 200/0.5 [15].

However, injury mechanisms remain unclear but the oncotic force of the fluid replacement solutions seems also to be important. When the oncotic pressure exceeds the hydraulic pressure of glomerular filtration it may suppress urine output [16]. Furthermore, glomerular filtration of hyperoncotic molecules from colloids causes hyper viscose urine and a stasis of tubular flow, resulting in obstruction of tubular lumen [17]. Recently, the occurrence of renal adverse events after resuscitation of shock in 822 ICU patients using crystalloids, hypooncotic (gelatin and 4% albumin) and hyperoncotic colloids (HES and dextran), and hyperoncotic 20% albumin was investigated in an observational multicenter cohort study [18]. The occurrence of renal adverse events was more frequent in both hyperoncotic groups compared to crystalloids and isooncotic colloids, suggesting that the hyperoncocity was more relevant than the nature of the product itself. Also in our study, the COP was significantly higher in the 10% HES200 and 4% gelatin groups after 12 h of sepsis compared to 6% HES130 and RAc. This might be one of the potential reasons for the pronounced kidney injury in the 10% HES200 group. However, as isooncotic 6% HES 130/0.42 (*in vitro *COP 37.8 mmHg) and hyperoncotic 10% HES 200/0.5 (*in vitro *COP 80 to 85 mmHg) were compared, it cannot be differentiated whether the oncotic force, the molecular weight, the degree of molar substitution, molecular size, the solvent for colloid solution, or a combination of these were determining factors of the HES-induced adverse effects on renal function and structure.

HES is partly eliminated by the reticuloendothelial system, and a dose dependent uptake in macrophages, endothelial and epithelial cells was detected [19]. Additionally, tissue deposition of HES was found in the kidneys. The HES containing vacuoles in the kidneys are called osmotic-like lesions (OL). This tissue deposition of HES seems to be transitory and dose-dependent [19,20]. In our investigation we found OL elevated in all colloid groups achieving a significant level in the 4% gelatin group.

In the renal histological analysis, we found in the 10% HES200 group significantly elevated acute tubular necrosis and interstitial bleeding compared to the other groups, indicating tubular injury, thereby explaining the increased NAG and reduced creatinine clearance.

In an *ex vivo *renal perfusion model, the administration of HES 130 and HES 200 solutions induced a significant decrease in diuresis and creatinine clearance compared with Ringer's lactate (RL). Interestingly, 10% HES 200/0.5 was associated in this experimental setting with significant elevated inflammatory response in tubular monocytes. This pronounced pro-inflammatory effect of 10% HES 200/0.5 compared with 6% HES 130/0.42 caused more tubular damage than 6% HES 130/0.42 and RL [12]. In our present *in vivo *study, 10% HES200 in comparison to 6% HES130, 4% gelatin and RAc induced higher inflammatory response as suggested by higher Il-6, IL-10 and TNF-α levels, which is in accordance with our previous data. This increased inflammatory response in the 10% HES200 group may be another explanation for the pronounced kidney injury. In line with these findings, Murugan *et al*. demonstrated a context between elevated immune response and kidney injury [21].

Lv *et al*. showed in endotoxaemic rats that 6% HES200 in comparison to 0.9% saline reduced the intestinal production of proinflammatory cytokines, including TNF and IL-6 [22].

The acetate seems not to play a role in the inflammatory modulation. Davies *et al*. found in a study of 30 adult patients undergoing cardiopulmonary bypass for elective cardiac surgery that the use of acetate buffered prime solutions for cardiopulmonary bypass result in supra-physiological concentrations of acetate. Nonetheless, there was no difference in interleukin-6 release compared to bicarbonate-balanced fluid [23].

Hoffmann *et al*. demonstrated that HES 130 is effective to prevent LPS-induced leukocyte adherence, to attenuate LPS-induced capillary perfusion failure, and to reduce LPS-induced macromolecular leakage indicating even an anti-inflammatory potential of HES 130 [24].

### Balanced solutions and acid-base balance

The concept of balanced fluid replacement and its relevance with respect to mortality remains unclear. So far, only limited data are available and to the best of our knowledge, the present study is the first one to investigate balanced fluid replacement solutions in a two-hit model of hemorrhagic and septic shock. Resuscitation using RAc in experimental hemorrhagic shock could attenuate acidosis better than 0.9% saline solution, resulting in an improved survival rate [25]. Kellum *et al*. demonstrated in septic rats that the use of a balanced Hextend solution in comparison with 0.9% saline solution was associated with improved acid balance and even survival [10]. In line with Kellums data, we found that using 6% HES 130/0.42 in RAc and RAc resulted in significantly less negative base excess than resuscitation using 10% HES 200/0.5 in 0.9% saline. The administered amount of the latter was less compared to RAc. This result indicates that solvent for colloid solutions may be of relevance in resuscitation of shock in order to avoid further acid-based derangements. Interestingly, we could not find any differences in the concentration of chloride and sodium in the blood, except for a significantly lower chloride level in the 4% gelatin group at study end, most likely due to the fact that 4% gelatin contains less chloride than RAc (4% gelatin 85 mmol/l; RAc 112 mmol/l). This is also reflected in a significantly higher calculated anion gap in the 4% gelatin group; the anion gap did not change in all other groups compared to baseline. Thus, the use of 0.9% saline as a solvent was not associated with unwanted electrolyte disturbances in our model.

Furthermore, it was not possible to prevent metabolic acidosis in the 10% HES 200 group with no change in the anion gap compared to baseline. The metabolic acidosis in the 10% HES 200/0.5 group may be in part explained by the elevated chloride levels (111 ± 2 mmol/l) due to the carrier solution. In addition, acetate is metabolized to HCO_3_^- ^in the other solutions and is acting as a buffer *in situ*.

### Limitations

A limitation of our model may be the experimental duration of 19 hours. On the other hand, Rivers *et al*. have shown that early goal-directed fluid resuscitation in patients with severe sepsis and septic shock provides significant benefits related to outcome in the first six hours after admission to an accident and emergency hospital (A&E) [2].

We chose CVP as our target for the infusion of the fluid replacement solutions according to the Surviving Sepsis Campaign: International guidelines for management of severe sepsis and septic shock [3]. Nevertheless there are several studies in critically ill patients [26,27] suggesting that ITBV is a valuable preload indicator. In our study, the data of ITBV suggest sufficient fluid status until the end of the experiment.

Another potential confounding factor may be gender differences in the inflammatory response and survival in sepsis [28]. Ovariectomized mice had a higher death rate than proestrus mice after cecal ligation and puncture [29]. This potential difference could not be addressed in our study because we used female pigs only.

## Conclusions

Despite similar maintenance of macrocirculation 6% hydroxyethylstarch 130/0.42 and Ringer's acetate significantly preserve renal function and attenuate tubular damage better than 10% hydroxyethylstarch 200/0.5 in saline.

## Key messages

• After resuscitation there was no difference in systemic hemodynamics comparing the investigated solutions.

• Ten percent HES 200/0.5 induced significant increased tubular damage compared to Ringers's acetate and 6% HES 130/0.42.

• Ten percent HES 200/0.5 induced significant renal impairment compared to Ringers's acetate and 6% HES 130/0.42.

## Abbreviations

10% HES200: 10% hydroxyethylstarch 200/0.5 in saline; 4% gelatin: 4% gelatin in acetate; 6% HES 130: 6% hydroxyethylstarch 130/0.42; ATN: acute tubular necrosis; BL: baseline; BW: body weight; CFU: colony forming units; CO: cardiac output; COP: colloid osmotic pressure; CrCl: Creatinine clearance; CVP: central venous pressure; DO_2_, systemic oxygen delivery; GSl: glomerulo sclerosis; Hct: hematocrit; HES: hydroxyethyl starch; Ibl: interstitial bleeding; IL-10: Interleukin 10; IL-6: Interleukin 6; ITBV: Intrathoracic blood volume; MAP: mean arterial pressure; MPAP: mean pulmonary artery pressure; NAG: N-acetyl- beta-D-glucosamidase; OE: oxygen extraction ratio; OL: osmotic-nephrosis like lesions; PAOP: pulmonary artery occlusion pressure; PEEP: positive end-expiratory pressure; Rac: Ringer's acetate; RBF: Renal blood flow; RL: Ringer's lactate; SD: standard deviation; SHAM: sham-treated group; TNF-α: tumor necrosis factor alpha; VISEP: efficacy of fluid substitution and insulin therapy in severe sepsis.

## Competing interests

This investigation was funded by a restricted grant from Serumwerke Bernburg, Bernburg, Germany. Serumwerke Bernburg holds the patents of several resuscitation solutions. TPS has received travel grants from BBraun and Serumwerke Bernburg. LH has received lecture fees from BBraun. GM has received honoraria for consulting or lecturing, and restricted research grants from the following companies: BBraun, Edwards Life Sciences, Serumwerke Bernburg, Hutchinson Technology, Baxter. All the other authors declare that they have no competing interests.

## Authors' contributions

TPS made substantial contributions to the conception and design of the study, acquisition of data, analysis and interpretation of data, and wrote the manuscript. TS participated in the design of the study, performed statistical analysis and helped to draft the manuscript. LH, MS and FH made substantial contributions to the design of the study and the acquisition of data. WP made substantial contributions to conception and design of the study and provided the bacterial clots. GM made substantial contributions to conception and design of the study, interpretation of data and wrote the manuscript. All authors read and approved the final manuscript for publication.

## Supplementary Material

Additional file 1 **Histological analysis of an animal kidney treated with 10%HES200**. The Sections (4 μm) were cut and stained with hematoxylin and eosin. Osmotic-nephrosis like lesions are widespread and marked with an arrow. Original magnification: × 200. Click here for file

Additional file 2 **Histological analysis of an animal kidney SHAM treated**. The Sections (4 μm) were cut and stained with hematoxylin and eosin. Osmotic-nephrosis like lesions are very rare. Original magnification: × 200. Click here for file

Additional file 3 **Histological analysis of an animal kidney treated with10% HES200**. The Sections (4 μm) were cut and stained with hematoxylin and eosin. Interstitial bleeding are marked with an arrow. Original magnification: × 200. Click here for file
