# Supplementary material for: Impairment of renal function using hyperoncotic colloids in a two hit model of shock: a prospective randomized study
Source: Crit Care. 2012 Jan 25;16(1):R16. doi: 10.1186/cc11161 (PMC3396252; doi:10.1186/cc11161)
Supplement: Additional file 3 — Histological analysis of an animal kidney treated with10% HES200. The Sections (4 μm) were cut and stained with hematoxylin and eosin. Interstitial bleeding are marked with an arrow. Original magnification: × 200. [file cc11161-S3.PDF]

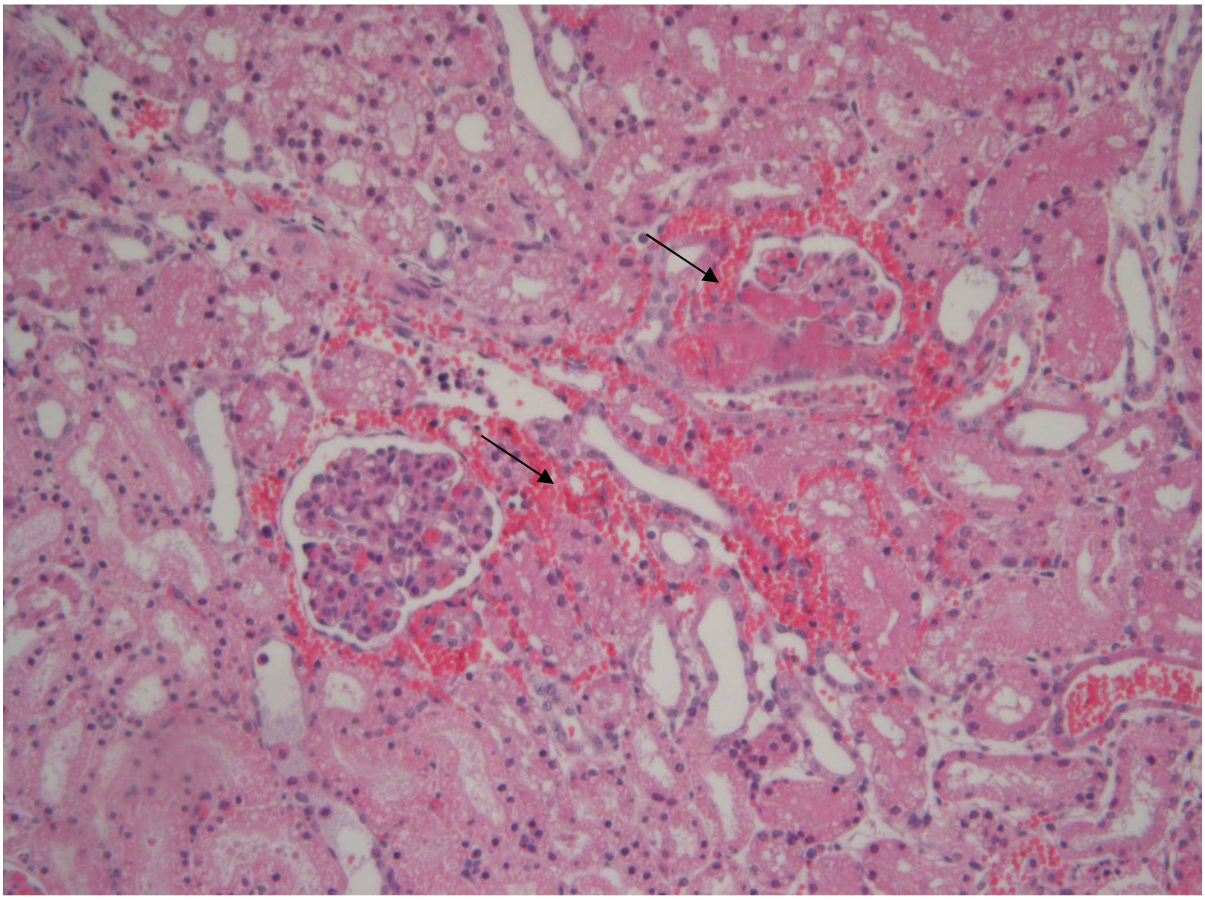

Picture 1: Histological analysis of an animal kidney treated with10% HES200. The Sections (4 µm) were cut and stained with hematoxylin and eosin. Interstitial bleeding are marked with an arrow. Original magnification: × 200.
